# Lightning ignition efficiency in Canadian forests

**DOI:** 10.1186/s42408-025-00376-1

**Published:** 2025-05-26

**Authors:** Sean C. P. Coogan, Alex J. Cannon, Mike D. Flannigan

**Affiliations:** 1 Department of Natural Resource Science, Thompson Rivers University, Kamloops, BC V2C 0C8 Canada; 2 Climate Research Division, Environment and Climate Change Canada, Victoria, BC V8N 1V8 Canada

**Keywords:** Fuel moisture, Lightning, Lightning-caused fires, Lightning efficiency, Lightning ignition, Wildfire

## Abstract

**Background:**

Lightning-caused fires have a driving influence on Canadian forests, being responsible for approximately half of all wildfires and 90% of the area burned. We created a climatology (2000–2020) of daily lightning efficiency (i.e., the ratio of cloud-to-ground lightning flashes to lightning-caused wildfires that occurred) over the meteorological summer for four ecozones and a subset of British Columbia (BC) ecoprovinces. We estimated lightning efficiency using data from the Canadian Lightning Detection Network and the Canadian National Fire Database. We used the ERA5 reanalysis as inputs for fuel moisture variables (i.e., Fine Fuel Moisture Code (FFMC), Duff Moisture Code (DMC), and Drought Code (DC)) from the Canadian Forest Fire Weather Index (FWI) System, as well as variables relating to the amount of precipitation and lightning flashes. We examined relationships between lightning efficiency, day-of-year, and the above variables using a combination of linear models, Spearman’s correlations, and Random Forest (RF) regression.

**Results:**

Lightning efficiency increased non-linearly (i.e., quadratic) over the summer in the Montane Cordillera Ecozone, and decreased linearly in the Boreal Plains and Boreal Shield West. Lightning efficiency in the Boreal Shield East showed a slight decline over the summer; however, this model was not significant. DMC and DC were more strongly correlated with lightning efficiency than FFMC in most zones. We ran RF regression both with and without DC (because of multicollinearity with day-of-year), and day-of-year, DMC, and DC (when present) were the most important variables for all ecozones, while results were more variable for the ecoprovinces.

**Conclusions:**

Lightning efficiency, and, thus, the probability of a lightning strike igniting a wildfire, changes over the summer and varies by region. Therefore, models predicting lightning-caused fire occurrence, or other similar applications involving lightning ignition, may benefit by accounting for seasonal lightning efficiency in addition to the traditional fuel moisture variables. Our work is generally consistent with findings from more localized studies relating to lightning-caused fires.

**Supplementary Information:**

The online version contains supplementary material available at 10.1186/s42408-025-00376-1.

## Resumen

**Antecedentes:**

Los incendios causados por rayos tienen una influencia preponderante en los bosques de Canadá, siendo responsables de aproximadamente la mitad de los incendios y del 90% del área quemada. Creamos una base de datos climatológica (2000–2020) sobre la “eficiencia diaria de los rayos” (i. e. la relación entre los flashes de rayos entre nubes y la tierra y los incendios que éstos realmente producían) en el verano meteorológico de cuatro ecozonas de y un subgrupo de ecoprovincias de la Columbia Británica (BC). Estimamos la eficiencia de los rayos usando datos de la red canadiense de detección de incendios y de la base nacional de datos de incendios de Canadá. Usamos el re-análisis ERA5 como entrada de las variables de humedad (i. e. el código de Humedad del Combustible, FFMC), el Código de humedad del mantillo, DMC), y el código de sequía (DM), del Sistema Canadiense de Índice de Peligro de Incendios (FWI), como así también variables que relacionan la cantidad de precipitación y los flashes de rayos. Examinamos las relaciones entre la eficiencia de los rayos, el día y año de ocurrencia, y las variables arriba mencionadas, usando una combinación de de modelos lineales, Correlación de Spearman, y Regresión de Bosques al Azar (RF).

**Resultados:**

La eficiencia de los rayos se incrementó de manera no linear (i. e. cuadrática), durante el verano en la Ecozona de la Cordillera Montana, y decreció linealmente en las planicies boreales y en el oeste de las planicies que van desde la costa este a las rocallosas (*Boreal Shield West*). La eficiencia de los rayos en provocar incendios mostró una suave disminución durante el verano; sin embargo, este modelo no fue significativo. El DMC y el DC estuvieron más fuertemente relacionados con la eficiencia de los rayos que el FFMC en casi todas las zonas. Corrimos la regresión RF tanto con y sin el DC (debido a su múltiple colinearidad con el día del año); el día del año, el DMC y el DC (cuando estuvo presente), fueron las variables más importantes para todas las ecozonas, mientras que los resultados fueron más variables para las ecoprovincias.

**Conclusiones:**

La eficiencia de los rayos y, por lo tanto, la probabilidad de que un rayo inicie un incendio varía por regiones. Por esa razón, los modelos que predicen la causa de la ocurrencia de incendios por rayos, u otras aplicaciones similares que implican la ignición por rayos, puede beneficiarse teniendo en cuenta la eficiencia estacional de de los incendios por rayos, de manera adicional a las tradicionales variables de humedad del combustible. Nuestro trabajo es en general consistente con los resultados encontrados en estudios más localizados relacionados con los incendios causados por rayos.

## Background

Wildland fire is a common occurrence in Canada, where an estimated average of 1.96 Mha of land has burned per year in recent times (ca. 1959–2015; Hanes et al. 2019). During this period, approximately 50% of wildland fires were caused by lightning, and the remaining 50% were caused by people (Hanes et al. 2019). Lightning-caused fires, however, were responsible for a much larger percentage of burned area in Canada (~ 90%) than human-caused fires (Hanes et al. 2019). This disparity in area burned may be in part explained by the fact that human-caused fires, which often occur closer to population centers and infrastructure, tend to be more quickly detected and more aggressively actioned than lightning-caused fires (Stocks et al. 2002). Additionally, because thunderstorms are often spatially organized (Hadavi and Romanic 2024), lightning-caused fire starts can occur in clusters that can overwhelm fire management agencies (Podur and Wotton 2010; Coogan et al. 2022). Lightning-caused fires, however, are often left to burn in remote areas, because fire has many positive benefits for ecosystems that have evolved with it (Rowe and Scotter 1973; Tymstra et al. 2020).

There are several factors that influence whether a cloud-to-ground lightning strike will ignite a wildfire (Podur et al. 2003; Hessilt et al. 2022). One factor is fire weather, which has a direct influence on fuel moisture content and thus the receptivity of fuel to ignition (Wotton and Martell 2005; Abatzoglou et al. 2016). Fuel moisture has been shown to play an important role in lightning caused fires (Wotton and Martell 2005; Nadeem et al. 2020). In Canada, the Canadian Forest Fire Weather Index (FWI) System (Van Wagner 1987) fuel moisture indices including the Fine Fuel Moisture Code (FFMC), Duff Moisture Code (DMC), and Drought Code (DC) are widely used by management agencies and scientists. Previous research has found the DMC to often be the most important fuel moisture variable for lightning-caused fires (Wotton and Martell 2005; Nadeem et al. 2020) and it is often used an indicator of lightning fire potential by Canadian management agencies (Wotton et al. 2005). However, some research has indicated the FFMC and DC can also be important factors in some areas (Nash and Johnson 1996; Nadeem et al. 2020).

One relatively simple but useful approach to studying lightning-caused fires is to examine lightning ignition efficiency as defined by the ratio of lightning strikes to the number of wildfires (or vice versa) that occurred in an area (Meisner 1993). Currently, there has been relatively little research related to lightning ignition efficiency in Canada; however, the research that has been undertaken has led to interesting findings. For example, one study found that the moisture content of fine fuels played an important role in lightning efficiency in the Boreal forest (Nash and Johnson 1996). Another study found striking regional differences in lightning efficiency between adjacent sections of their study area in the Central Cordillera of British Columbia (BC) and Alberta which were separated by the continental divide (Wierzchowski et al 2002). In that study, the authors reported that, on average, there was 1 fire for every 50 lightning discharges in the BC portion of their study area, and one fire for every 1400 lightning discharges on the adjacent Alberta side.

In this study, we further investigate lightning efficiency in Canada by creating a climatology of lightning efficiency across broad but ecologically similar regions of Canada (i.e., ecozones) as well as a subset of smaller regions (i.e., ecoprovinces) in BC over the meteorological summer (June–August). Such an examination will give insight into the potential for lightning to ignite a fire during a particular time of the summer in a particular region. Given the spatiotemporal variation in the occurrence of both lightning and wildland fire across Canada (Hanes et al. 2019; Kochtubajda and Burrows 2020; Coogan et al. 2020), we expect there to be differences in lightning efficiency over the summer and between ecozones. We also investigate the importance of drivers associated with lightning efficiency, with a focus on the fuel moisture indices from the FWI System. We also examine variables relating to precipitation, cloud-to-ground lightning density, and dry lightning; these factors have also been identified as important for lightning-caused fire ignitions (Peterson et al. 2010; Kalashnikov et al. 2022). To our knowledge, a broad-scale investigation into the spatial and temporal variation in lightning efficiency has yet to be undertaken in Canada and could provide valuable information to researchers and wildfire management agencies involved in lightning-caused fire prediction.

## Methods

### Lightning and fire data

We used lightning data (2000–2020) collected through the Canadian Lightning Detection Network (CLDN) which was analyzed and quality controlled by Vaisala Inc. A comprehensive description of the CLDN can be found in Kochtubajda and Burrows (2020). We used cloud-to-ground lightning flash data to calculate lightning efficiency. A lightning flash consists of one to several strokes per flash (i.e., “multiplicity”). The dataset also contains information related to the polarity of lightning flashes (positive or negative) which we summarize in our results. For wildfire dates and locations, we used the Canadian National Fire Database (CNFDB) fire point data (available at https://cwfis.cfs.nrcan.gc.ca/datamart). The CNFDB is a compilation of wildland fire locations supplied by provincial, territorial and Parks Canada fire management agencies and is regularly used for spatiotemporal analyses of landscape-scale fire effects. We constrained our estimates of lightning ignition efficiency to the meteorological summer (i.e., June–August) when the majority of lightning strikes and lightning-caused fires occur in Canada (Coogan et al. 2020; Kochtubajda and Burrows 2020).

### Study area

**Fig. 1:**
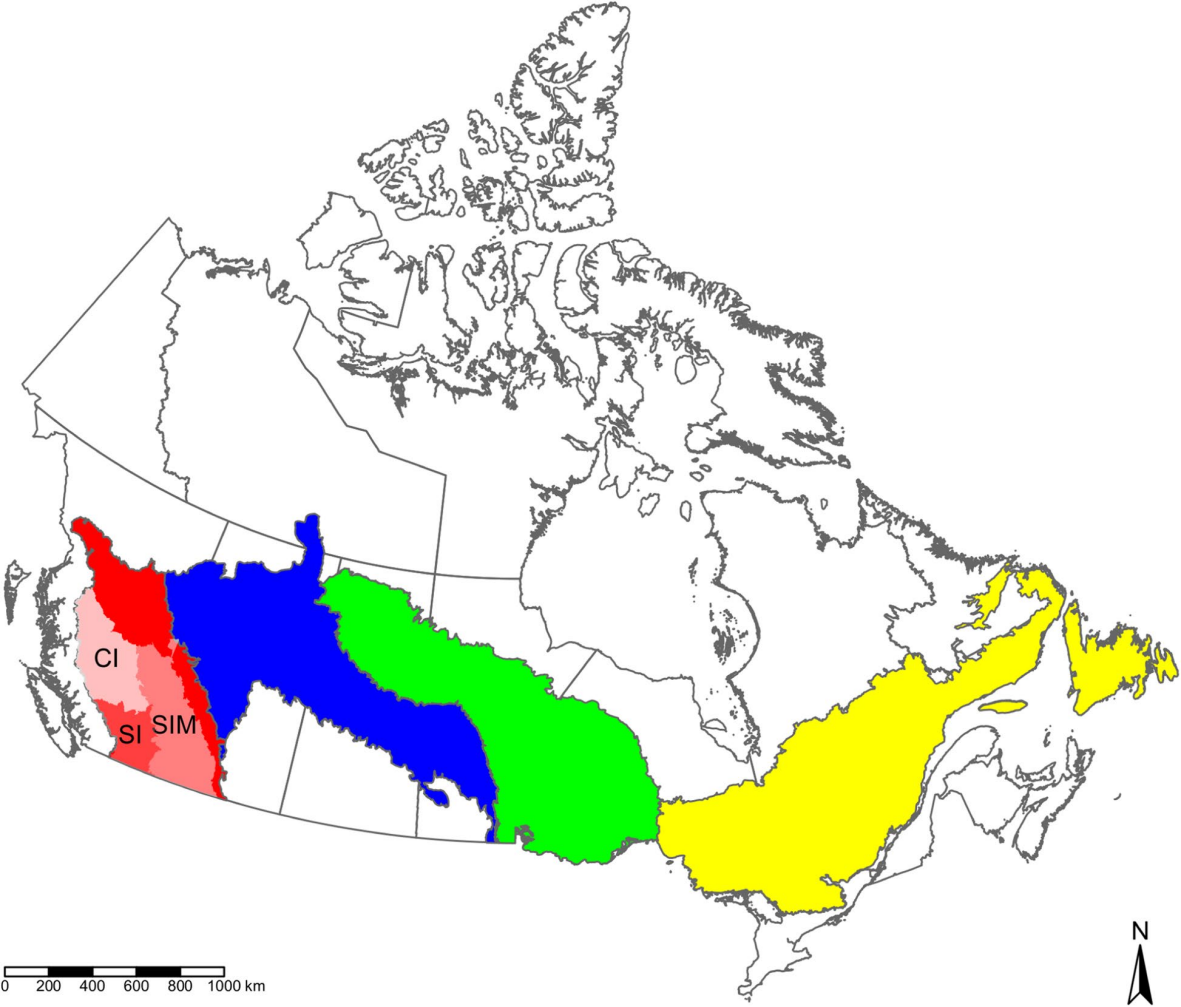
Lightning efficiency was estimated for the highlighted Canadian ecozones including, from west to east, the Montane Cordillera (red), Boreal Plains (blue), Boreal Shield West (green), and Boreal Shield East (yellow). Lightning efficiency was also estimated for a subset of ecoprovinces within the Montane Cordillera including the Southern Interior Mountains (SIM), Southern Interior (SI), and Central Interior (CI)

We used the ecozone classification from the Canadian Ecological Land Classification (Ecological Stratification Working Group 1995) to delineate broad areas (i.e., ecozones) for which to estimate lightning ignition efficiency. Ecozones represent large areas of very generalized ecological units characterized by similar biotic and abiotic factors (Marshall et al. 1999). We focused on the ecozones which had the greatest amount of fire occurrence data, including (from west to east) the Montane Cordillera (MC), Boreal Plains (BP), Boreal Shield West (BSW), and Boreal Shield East (BSE; Fig. 1). Note that the Boreal Shield Ecozone is often split into two sections (i.e., BSW and BSE) in Canadian wildfire research because of its large size and different fire regime characteristics (Stocks et al. 2002; Hanes et al. 2019).

The MC extends from north-central BC to the US border and includes the BC interior mountain ranges and valleys as well as the Alberta Foothills (Ecological Stratification Working Group 1995). The MC has the most diverse climate of Canadian ecozones as it contains areas that differ greatly in temperature, precipitation, and altitude including alpine tundra, dry grasslands, and dense conifer forests. The BP lies in part of the flat interior plains of Canada and stretches across portions of Manitoba and Saskatchewan and covers nearly two-thirds of Alberta (Ecological Stratification Working Group 1995). The climate is characterized by long, cold winters and short, warm summers, in part due to the Rocky Mountains to the west blocking moisture-laden air from the Pacific Ocean. The Boreal Shield Ecozone is the largest ecozone in Canada, extending from Saskatchewan to Newfoundland (Ecological Stratification Working Group 1995). The Boreal Shield climate is typically continental with short summers and long, cold winters. Areas in proximity to the Great Lakes and the Atlantic Ocean tend to be cooler in the summer due the moderating influence of these water bodies.

We also investigated lightning efficiency in three BC ecoprovinces that experienced the most lightning-caused fires including the Southern Interior (SI), Central Interior (CI), and Southern Interior Mountains (SIM; Fig. 1) to examine intra-ecozone variability in one of the regions. An ecoprovince is a subdivision of an ecozone, in this case the MC, which is characterized by major ecological assemblages (Marshall et al. 1999). The MC was selected for finer-scale investigation due to the distinctly different pattern in lightning ignition efficiency we observed compared to other ecozones (see Results). The province of BC has also experienced noteworthy fire seasons over the past decade with far-reaching impacts (e.g., smoke transport), and has been a region of concern over recent fire seasons.

### Lightning efficiency and modelling

We first converted the dates for both lightning flashes and fire starts to their corresponding day-of-the-year (DOY). We then summed across years (2000–2020) both the number of fires and number of lightning flashes that occurred on each DOY of the time series. We estimated lightning efficiency for each DOY by dividing the number of cloud-to-ground flashes that occurred by the number of lightning-caused fires that occurred, thereby producing a ratio of the number of flashes per fire that occurred on each DOY. In this metric of lightning ignition efficiency, a higher ratio of flashes to fires indicates lower efficiency — i.e., more lightning flashes occurred relative the number of fires for that DOY, suggesting that lightning was less likely to start an ignition — and vice versa. The time series was adjusted to account for effect of leap years (see Supplementary Material).

We characterized and contrasted the change in daily lightning efficiency over the summer by using the fitted values from linear regression models with DOY as the predictor variable (i.e., lightning efficiency ~ DOY), including the possibility of quadratic terms where those models performed better (based on adjusted R-squared and F-test values). We chose to use linear models for several reasons. Under the Gauss-Markov theorem, for example, ordinary least squares estimators are considered to be the best linear unbiased estimator (BLUE; Hansen 2022). Under this theorem, the model errors do not need to be normally distributed, which in our case was true for some regions (i.e., the BP, BSE, SI, and CI were right skewed; see model summary outputs in the Supplementary Material). The use of linear models consistently across ecozones and ecoprovinces also facilitated comparisons between them and helped to simplify the presentation of the relationship of lightning efficiency over time. It should be noted that we avoided transforming the data; however, we removed two large outliers from both the BSE and SI data set to improve the model generalizability, fit, and distribution of residuals. Linear models were created using the “lm” function in R version 4.1.0. (R Core Team 2020).

We also examined correlations between lightning efficiency and six potentially related variables. Brief explanations of the three aforementioned fuel moisture variables we examined are as follows: the FFMC is a numeric rating of the moisture content of cured fine fuels and surface litter; the DMC relates to the moisture content of organic layers of moderate depth that are loosely compacted; and the DC is a rating of the average moisture content of deep, compact organic layers. Meteorological variables (i.e., temperature, windspeed, relative humidity, and precipitation) from the ERA5 reanalysis (Hersbach et al. 2020) were used as inputs to create the FWI System fuel moisture variables following the procedure used in Jain et al. (2022). The daily value of each variable was averaged across the time series (2000–2020) for each DOY. We also examined a selection of other variables associated with lightning-caused fires including average precipitation (mm) and average number of lightning flashes for each DOY. Furthermore, we created a variable to examine the amount of average precipitation relative to lightning flashes that occurred for each day in each region (DRYL) by dividing precipitation by the number of flashes that occurred for each DOY. We examined correlations between the aforementioned variables and lightning efficiency using Spearman’s rho (R_s_), and, for qualitative purposes, we considered correlations to be high (> 0.7), moderate (0.4–0.69), and weak (0.1–0.39) based on broadly accepted categories (e.g., Akoglu 2018).

Finally, we created Random Forest (RF) regression models (Breiman 2001) to further evaluate which of the aforementioned covariates (including DOY) were the most important to lightning efficiency in the regions examined. We optimized the number of trees used in each model by using the number of trees that produced the lowest mean squared error (MSE). Models were created using the “randomForest” package (Liaw and Wiener 2002) in R version 4.1.0. (R Core Team 2020). To evaluate variable importance, we report both the percent increase in Mean Squared Error (%IncMSE) and Increasing Node Purity (IncNodePurity). In this case, the %IncMSE reflects the change in error that occurs when permuting a feature value; if permuting a feature does not increase the model error, then that feature is considered unimportant (and vice versa). Thus, higher values of %IncMSE indicate more important variables in this approach. The IncNodePurity uses the residual sum of squares (for regression) to indicate the change in the homogeneity of the groups created by the trees. When splitting the data, the data is partitioned by the attribute that results in the least impurity of the new nodes. Therefore, variables with higher IncNodePurity are considered to be more important in this approach. The variable importance from these two approaches can differ, however, which is why we opted to report both.

## Results

### Characteristics of lightning flashes and lightning-caused fires

**Fig. 2:**
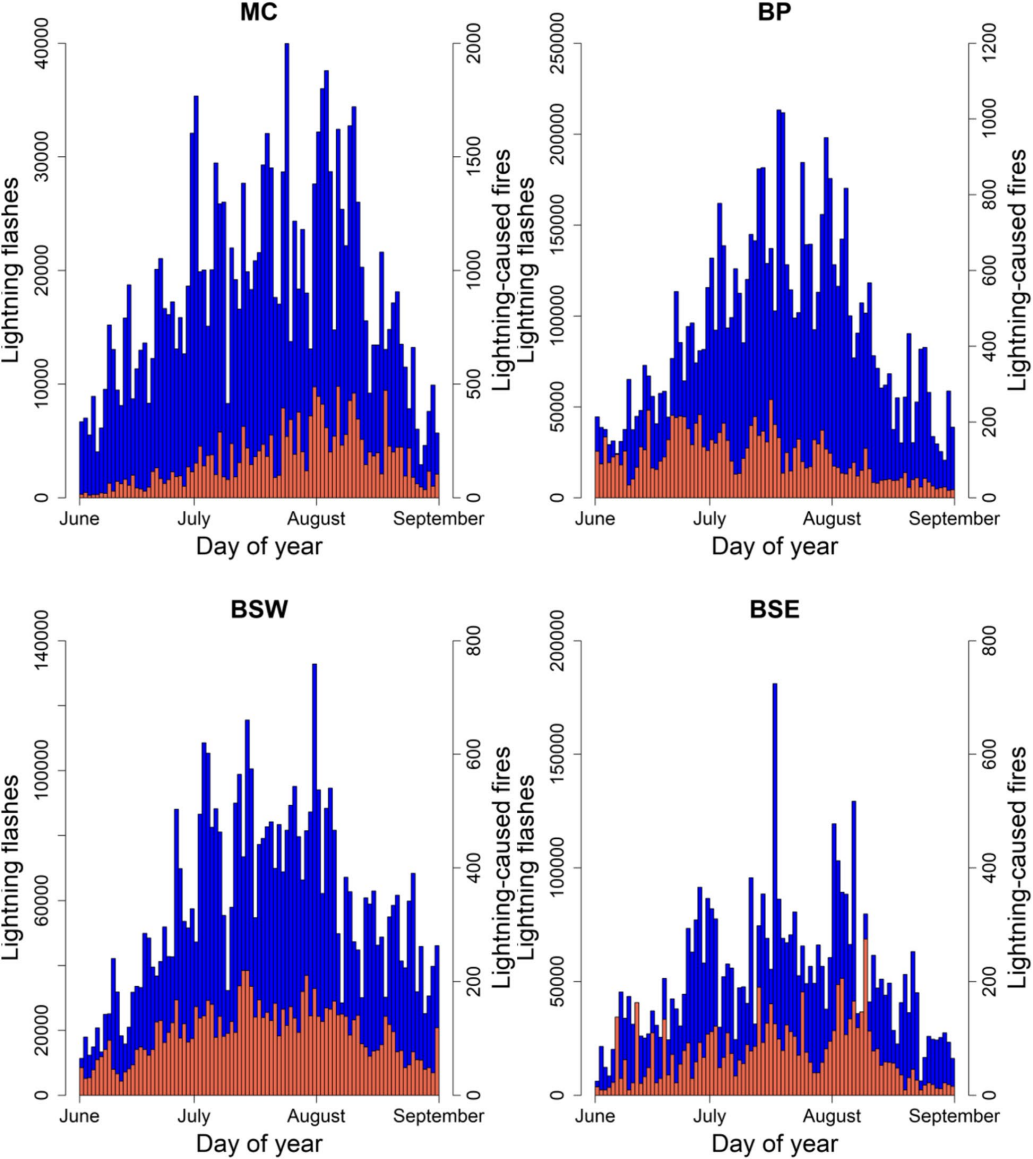
The daily total number of cloud-to-ground lightning flashes (blue bars, left *y*-axis) and lightning-caused wildfires (orange bars, right *y*-axis) that occurred in the Montane Cordillera (MC), Boreal Plains (BP), Boreal Shield West (BSW), and Boreal Shield East (BSE) Ecozones over the meteorological summer (June–August) from 2001 to 2020

For the MC, there was total of 1,640,577 lightning flashes recorded (3.4 flashes per km^2^). The average multiplicity was 2.0 (range 1–15), 84.5% of flashes had a negative charge, and 15.5% had a positive charge. There was a total of 15,659 lightning-caused fires (0.03 fires per km^2^). In general, the number of flashes rose over June and July peaking around late-July to early-August and then declining (Fig. 2). The pattern for fires was similar to that observed for flashes.

For the BP, there was a total of 8,272,056 lightning flashes recorded (11.6 flashes per km^2^). The average multiplicity per flash was 2.3 (range 1–15), 80.9% of flashes had a negative charge, and 19.1% had a positive charge. There was a total of 9901 lightning-caused fires (0.01 fires per km^2^). In general, the number of flashes showed a curved shape which peaked around mid- to late-July (Fig. 2). However, the number of fires was highest in mid-June to mid-July, and declined in August.

For the BSW, there was a total of 5,242,235 lightning flashes recorded (6.5 flashes per km^2^). The average multiplicity per flash was 1.8 (range 1–15), 80.5% of flashes had a negative charge, and 19.5% had a positive charge. There was a total of 10,091 lightning-caused fires (0.01 fires per km^2^). In general, the number of flashes and fires both displayed a curved shape which peaked around mid-July (Fig. 2).

For the BSE, there was a total of 4,620,023 lightning flashes recorded (4.4 flashes per km^2^). The average multiplicity per flash was 1.9 (range 1–15), 86.8% of flashes had a negative charge, and 13.2% had a positive charge. There was a total 6799 fires (0.006 fires per km^2^). In general, the number of flashes and fires both rose to peak around early August then declined (Fig. 2). However, there appeared to be more variability in the days in which large numbers of fires occurred relative to the number of flashes that occurred.

### Lightning ignition efficiency as a function of DOY

All model predictions for lightning efficiency are summarized in Table 1, while descriptions of the models are given below. In Table 1, we included a standardized density of both flashes and fires for each region (per km^2^) to facilitate comparisons between the ecozones and ecoprovinces, all of which differ in area. All R generated model summaries are presented in the Supplementary Material (Figs. S1 – S7).

**Table 1:** Summary of linear model predictions of lightning efficiency (i.e., the ratio of cloud-to-ground lightning flashes per lightning-caused fire) for specified Canadian ecozones and British Columbia ecoprovinces

Region	Minimum predicted efficiency (flashes per fire)	Maximum predicted efficiency (flashes per fire)	Flashes per km^2^	Fires per km^2^
*Ecozone*				
Montane Cordillera	373	81	3.4	0.03
Boreal Plains	1705	388	11.6	0.01
Boreal Shield West	632	449	6.5	0.01
Boreal Shield East	933	777	4.4	0.006
*Ecoprovince*				
Southern Interior Mountains	400	51	4.2	0.06
Southern Interior	145	36	2.7	0.06
Central Interior	279	110	4.6	0.05

**Fig. 3:**
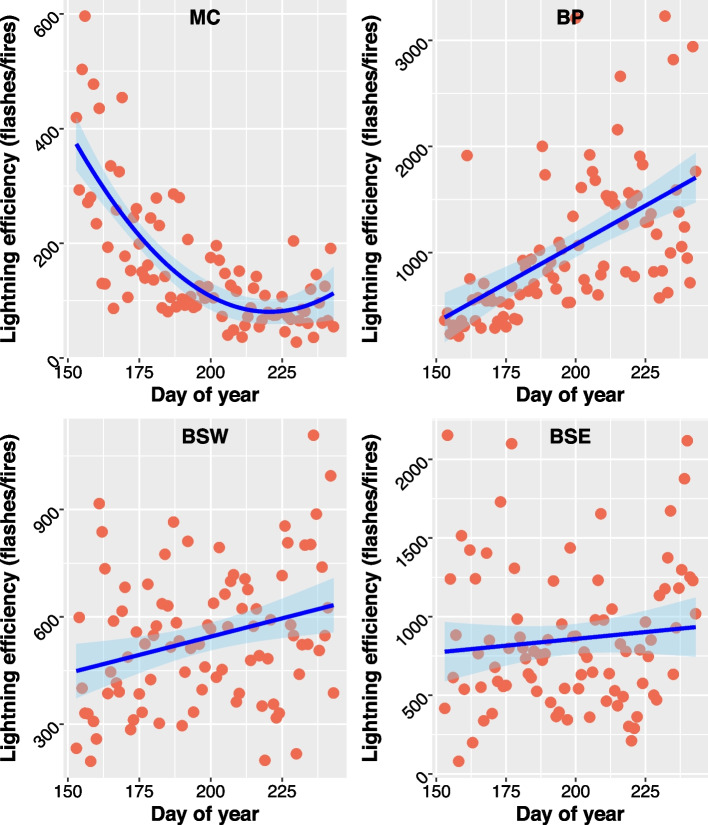
Daily lightning efficiency (i.e., the ratio of cloud-to-ground lightning flashes per lightning-caused fire) for the Montane Cordillera (MC), Boreal Plains (BP), Boreal Shield West (BSW), and Boreal Shield East (BSE) Ecozones over the meteorological summer (June–August; 2001–2020). Note that, as the ratio of flashes to fires decreases, lightning efficiency increases (i.e., there are fewer flashes per fire). Trendline and 95% confidence interval were fit using linear models (MC adj. R_2_ = 0.5608; BP adj. R_2_ = 0.3185; BSW adj. R_2_ = 0.0704; BSE adj. R_2_ = − 0.0011)

The ratio of flashes to fires in the MC decreased (increased in efficiency) non-linearly (i.e., quadratic) from June to early August at which point the ratio levelled off and began to increase again around mid-August (decreased in efficiency; Fig. 3). The model with DOY expressed as a quadratic variable (Lightning Efficiency ~ DOY + DOY^2^; adj. R^2^ = 0.5608; F(2,88) = 58.47, *p* = < 2.2e − 16) better fit the data than a univariate model (Lightning Efficiency ~ DOY; adj. R^2^ = 0.4428) based on adj. R^2^. The model predicted the highest efficiency to be 81 flashes per fire on 09 August. In the BP, the ratio of flashes to fires increased (efficiency decreased) over the summer (Lightning Efficiency ~ DOY; adj. R^2^ = 0.3185; F(1,89 = 42.48, *p*-value = 4.17e − 09)). In the BSW, the ratio of flashes to fires also increased (efficiency decreased) over the summer; however, the model (F(1,89) = 7.82, *p*-value = 0.0063) had a low adj. R^2^ (0.0704). The scatter of lightning efficiency data points showed a lot of variation over the summer in this ecozone (Fig. 3). The ratio of flashes to fires also increased (efficiency decreased) over the summer in the BSE (Fig. 3); however, the coefficient for the DOY variable was not significant (*p*-value = 0.344). The model also displayed poor fit to the data and was not significant even after removing the two large outliers (adj. R^2^ = − 0.0011; F(1,87) = 0.9069, *p*-value = 0.3436). The model that included the two outliers that were removed from the BSE analysis was similar but did not perform as well (Supplemental Material Figs. S4B and S8).

**Fig. 4:**
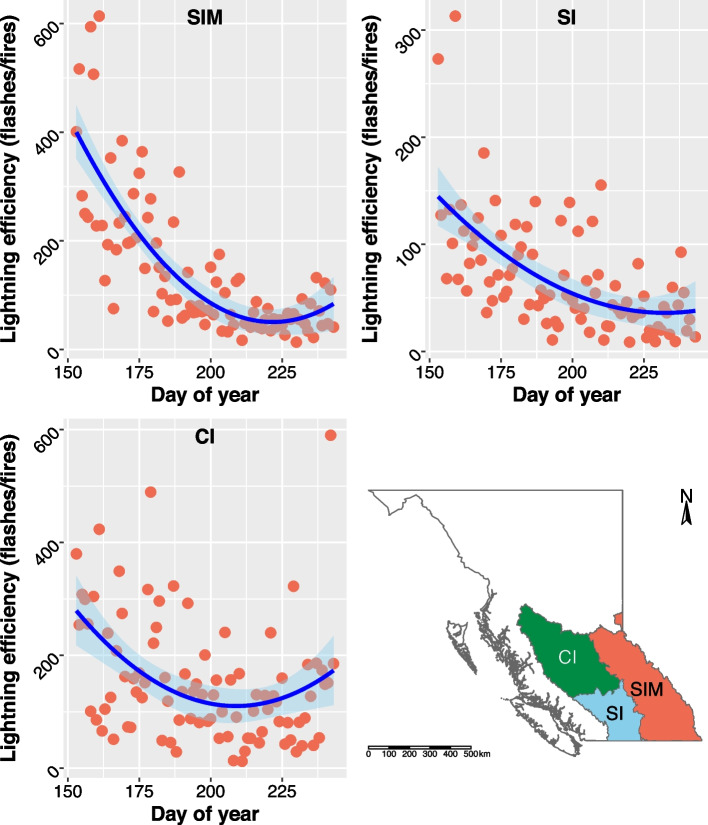
Daily lightning efficiency (i.e., the ratio of cloud-to-ground lightning flashes per lightning-caused fire) for the Southern Interior Mountains (SIM), Southern Interior (SI), and Central Interior (CI) Ecoprovinces over the meteorological summer (June–August; 2001–2020). Note that, as the ratio of flashes to fires decreases, lightning efficiency increases (i.e., there are fewer flashes per fire). Trendline and 95% confidence interval were fit using linear models (SIM adj. R^2^ = 0.6231; SI adj. R_2_ = 0.3582; CI adj. R_2_ = 0.1572). The ecoprovinces are shown in the bottom right panel for reference

For the BC ecoprovinces (Fig. 4), we found that quadratic models better fit the data, similar to the MC, where the ratio of flashes to fires decreased (increased in efficiency) over the summer to early- to mid-August including the SIM (adj. R^2^ = 0.6231; F(2,88), *p*-value = 2.2e − 16), SI (adj. R^2^ = 0.3582; F(2,86), *p*-value = 1.946e − 9)), and CI (adj. R^2^ = 0.1572; F(2,88), *p*-value = 0.0002). The SI had the highest predicted lightning efficiency, followed by the SIM and CI (Table 1). Furthermore, the trendline for the SI did not show a prominent increase in the ratio of flashes to fires (decrease in efficiency) in mid-August, as was present in the SIM, CI, and MC; instead, it seemed to remain more stable at its most efficient during this period. The model that included the two outliers that were removed from the SI analysis was similar to that reported here but did not perform as well (Supplemental Material Figs. S6B).

### Fuel moisture over the summer

**Fig. 5:**
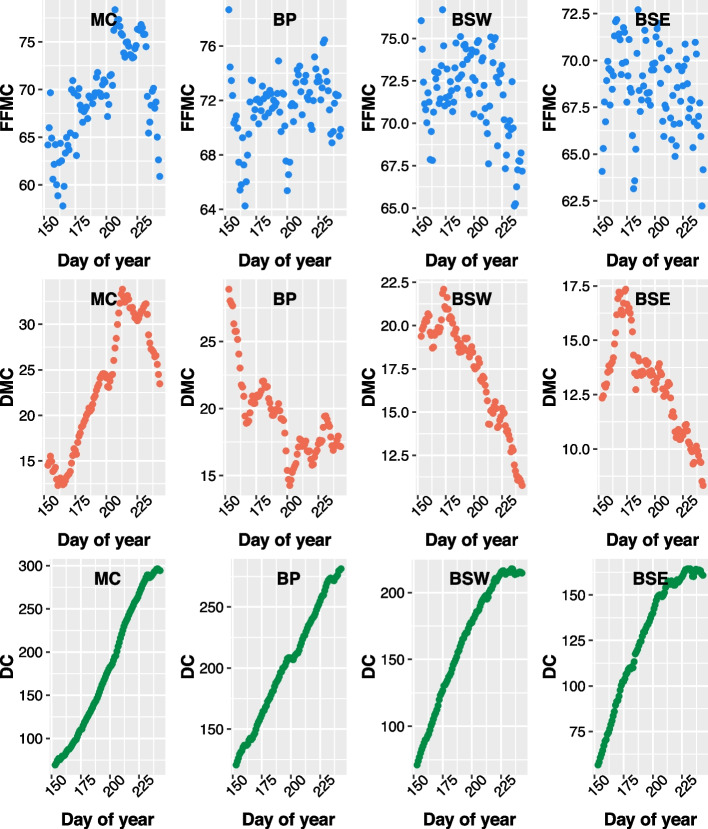
Average daily values (2001–2020) for the Fine Fuel Moisture Code (FFMC; top row), Duff Moisture Code (DMC; middle row), and Duff Code (DC; bottom row) for the Montane Cordillera (MC), Boreal Plains (BP), Boreal Shield West (BSW), and Boreal Shield East (BSE) Ecozones over the meteorological summer (June–August)

The FFMC displayed the most variation over the summer compared to the DMC and DC in the ecozones (Fig. 5); however, the clearest pattern for FFMC was found in the MC, which resembled the pattern for DMC in that ecozone. Average DMC values in the MC tended to increase from a low point in mid-June to a high point in early August, after which it declined again. In the BP, DMC declined from its highest point in early June to its lowest in late July. Both the BSW and BSE saw DMC increase initially in June to decrease to its lowest point at the end of August. DC showed strong consistent increases from June across all ecozones. DC levelled off somewhat at the end of August in the Boreal Shield West and East. Fuel moisture patterns for the BC ecoprovinces (i.e., SIM, SI, and CI) were similar to those of the MC and are displayed in the Supplementary Material (Fig. S9).

### Correlations

The FFMC was moderately negatively correlated with the ratio of flashes to fires in the MC and the SIM, SI, and CI, while being weakly correlated in the BSW, and uncorrelated in the BP and BSE (Table 2). The DMC was highly negatively correlated with the ratio of flashes to fires in the SIM, moderately correlated in the MC, BP, SI, and CI, and weakly correlated in the BSW and BSE. The negative correlation between DMC and lightning efficiency indicates that as DMC increases (becomes drier) the number of lightning flashes per fire decreases (becomes more efficient). The DC was highly negatively correlated with the ratio of flashes to fires in the SIM and MC, moderately correlated in the SI, and weakly correlated in the CI. Positive correlations with DC (suggesting decreasing efficiency as DC increased).

**Table 2:** Correlations (Spearman’s rho (R_s_)) between lightning efficiency (i.e., the ratio of cloud-to-ground lightning flashes per lightning-caused fire) and average daily FFMC (Fine Fuel Moisture Code), DMC (Duff Moisture Code), DC (Duff Code), PRECIP (precipitation in mm), LDENS (number of cloud-to-ground lightning flashes), and DRYL (the ratio of PRECIP to DRYL). Highly correlated (R_s_ > 0.7) variables are shown in boldfont, and moderately correlated variables (R_s_ = 0.4–0.69) are shown in italicfont. *P*-values are shown in parentheses

Region	FFMC	DMC	DC	PRECIP	LDENS	DRYL
*Ecozone*						
Montane Cordillera	* − 0.51* (3.7e* − *7)	* − 0.69* (< 2.2e* − *16)	* − ***0.70** (< 2.2e* − *16)	*0.44* (1.5e* − *5)	* − *0.19 (0.0677)	0.39 (0.0002)
Boreal Plains	* − *0.03 (0.7723)	* − 0.65* (< 2.2e* − *16)	*0.66* (< 2.2e* − *16)	< 0.01 (0.9597)	*0.48* (2.4e* − *6)	* − *0.33 (0.0015)
Boreal Shield West	* − *0.28 (0.0073)	* − *0.29 (0.005)	0.27 (0.0093)	*0.49* (1.4e* − *6)	0.32 (0.0021)	0.16 (0.1311)
Boreal Shield East	* − *0.09 (0.3903)	* − *0.15 (0.1627)	0.10 (0.3362)	* − *0.06 (0.5593)	0.12 (0.2559)	* − *0.15 (0.1634)
*Ecoprovince*						
Southern Interior Mountains	* − 0.69* (2.2e* − *16)	* − ***0.76** (2.2e* − *16)	* − ***0.75** (2.2e* − *16)	*0.61* (2.2e* − *16)	* − *0.30 (0.004)	*0.58* (2.92e* − *9))
Southern Interior	* − 0.52* (1.8e* − *7)	* − 0.58* (3.1e* − *9)	* − 0.60* (1.1e* − *10)	*0.52* (2.4e* − *7)	* − *0.05 (0.671)	0.39 (0.0002)
Central Interior	* − 0.52* (7.9e* − *5)	* − 0.42* (4.1e* − *5)	* − *0.32 (0.002)	0.33 (2.4e* − *7)	* − *0.22 (0.034)	0.36 (0.0006)

**Fig. 6:**
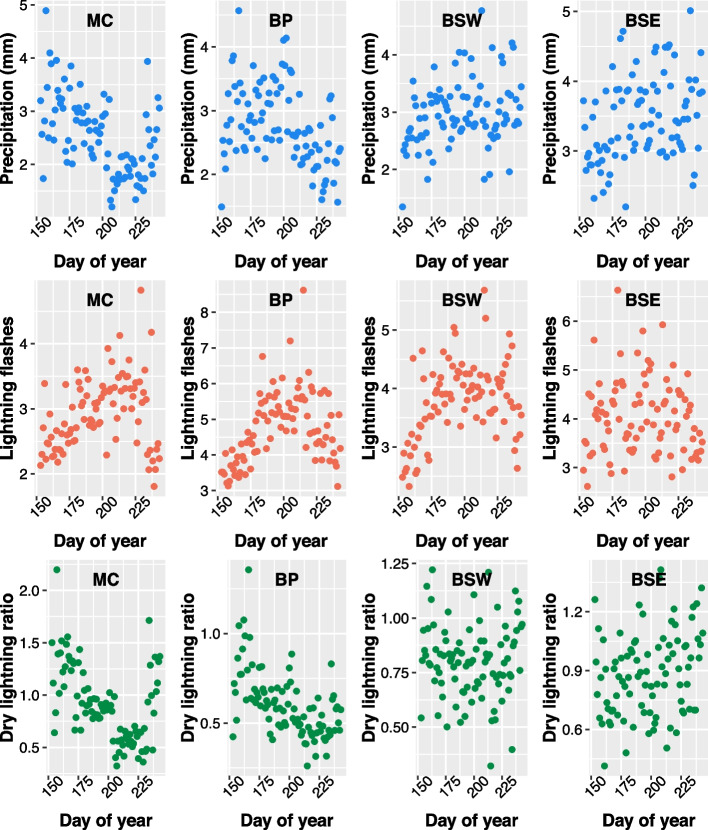
Average daily values (2001–2020) For precipitation (mm; top row), number of cloud-to-ground lightning flashes (middle row), and dry lightning ratio (i.e., the ratio of precipitation to flashes; bottom row) for the Montane Cordillera (MC), Boreal Plains (BP), Boreal Shield West (BSW), and Boreal Shield East (BSE) Ecozones over the meteorological summer (June–August)

Average daily precipitation was moderately positively correlated with the ratio of flashes to fires in the BSW, MC, SIM, and SI, was weakly correlated in the CI, and was uncorrelated in the BP and BSE (Table 2). Positive correlations suggest that as precipitation increases lightning becomes less efficient at starting a fire. For the average number of cloud-to-ground flashes, there were weak negative correlations with the ratio of flashes to fires in the MC, SIM, and CI — negative correlations suggest increased lightning efficiency with increasing lightning occurrence. However, there were positive correlations (suggesting decreased efficiency with increasing lightning occurrence) in the BP (moderate), BSW (weak), and BSE (weak). There was no correlation between the average number of cloud-to-ground flashes and the ratio of flashes to fires in the SI. DRYL was moderately positively correlated with the ratio of flashes per fire in the SIM, weakly correlated in the MC, SI, CI, and BSW, where a positive correlation between suggests that as the ratio of precipitation to lightning flashes increased (i.e., lightning in a region was generally accompanied by more rain) the number of flashes per fire increased (became less efficient). There was, however, a weak negative correlation in the BP and BSE. The seasonal patterns in average daily precipitation, lightning flashes, and DRYL for ecozones are shown in Fig. 6, and for ecoprovinces in Fig. S10.

### Random Forest regression models

**Fig. 7:**
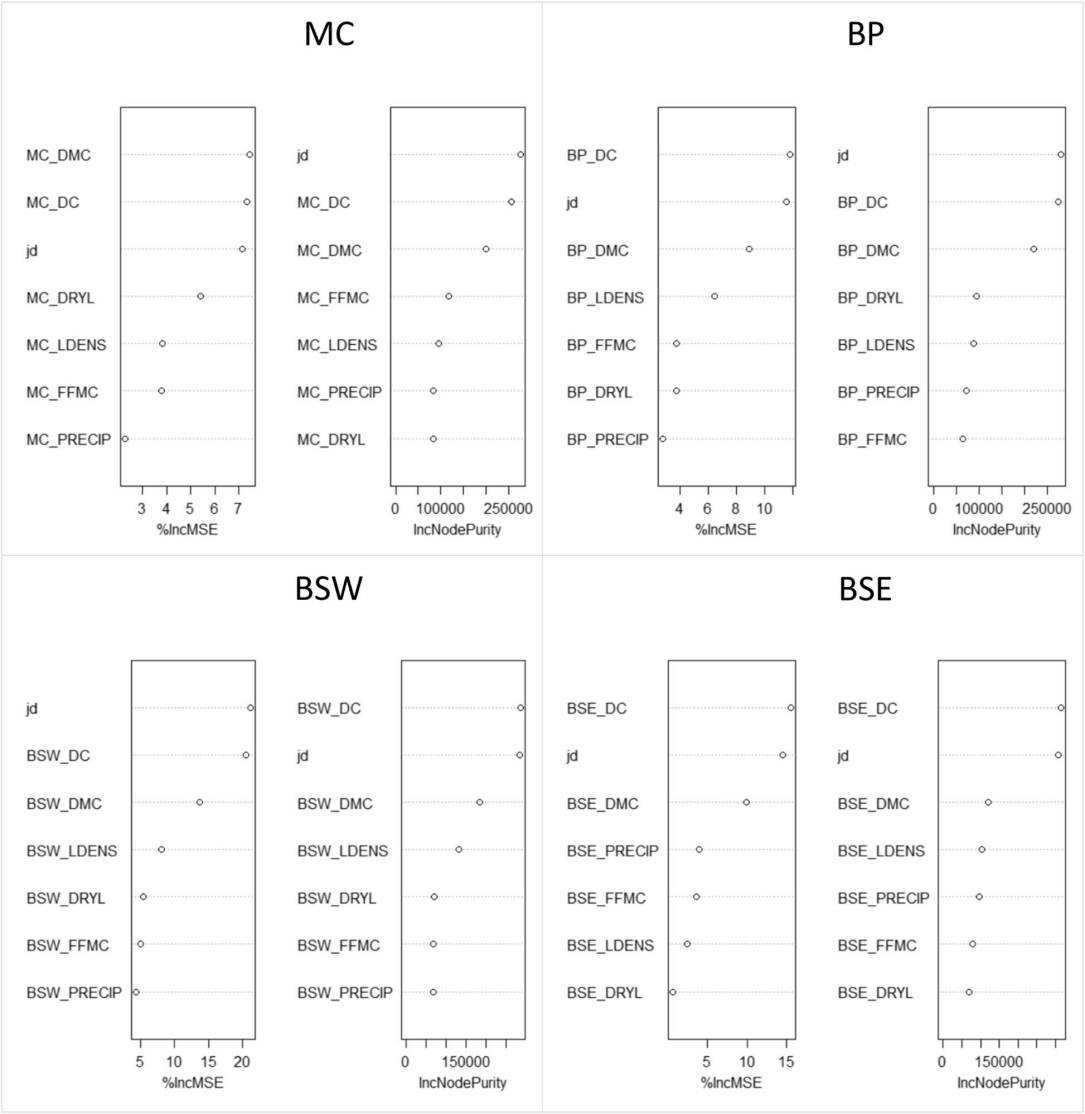
Variable importance for Random Forest regression models, including both the percent increase in mean squared error (%IncMSE) and increasing node purity (IncNodePurity) metrics, for the Montane Cordillera (MC), Boreal Plains (BP), Boreal Shield West (BSW), and Boreal Shield East (BSE) ecozones. Variables listed include the Drought Code (DC), Duff Moisture Code (DMC), Fine Fuel Moisture Code (FFMC), precipitation (PRECIP), number of lightning flashes (LDENS), ratio of precipitation to lightning flashes (DRYL), and day-of-year (jd)

**Fig. 8:**
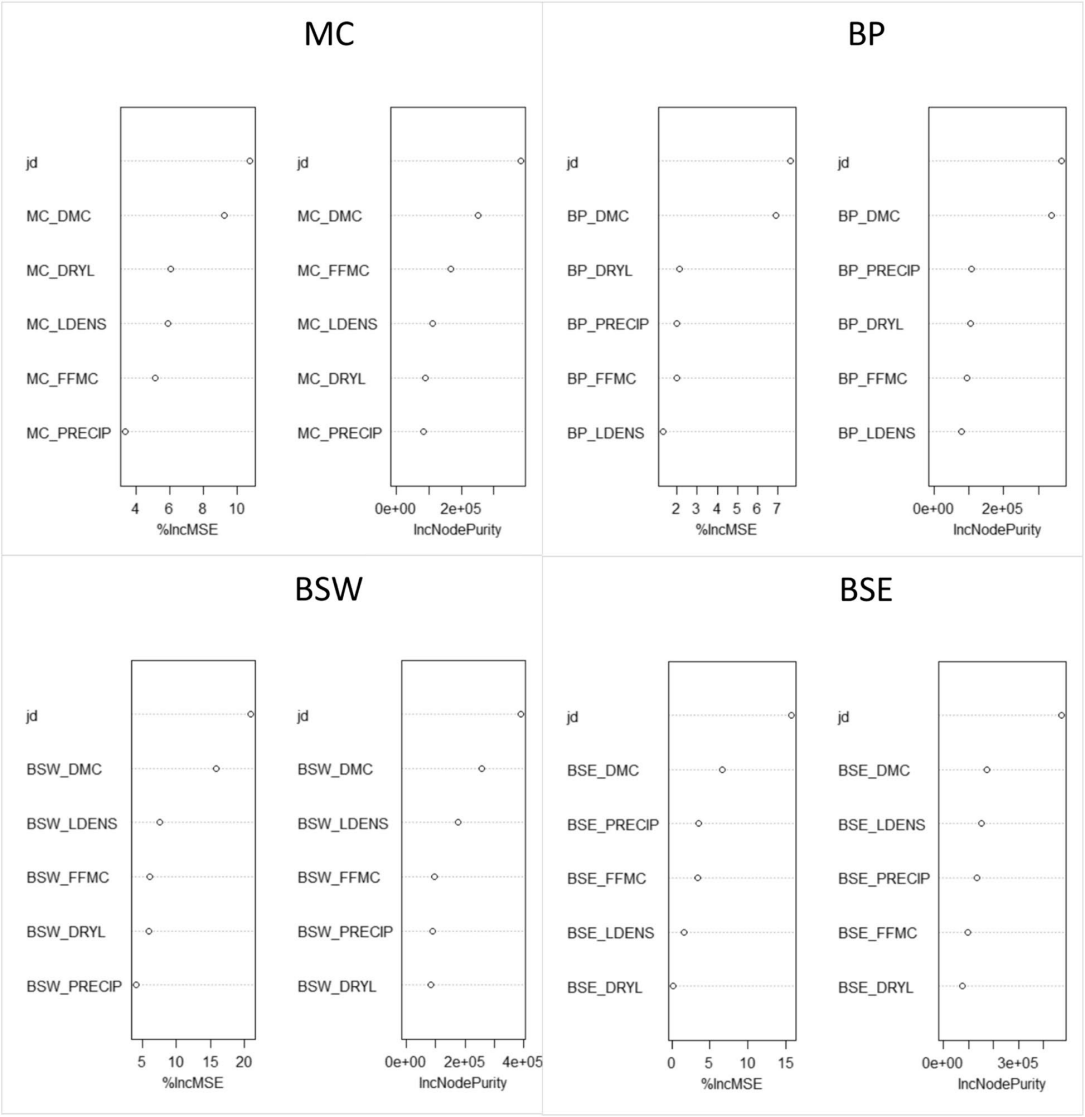
Variable importance for Random Forest regression models, including both the percent increase in mean squared error (%IncMSE) and increasing node purity (IncNodePurity) metrics, for the Montane Cordillera (MC), Boreal Plains (BP), Boreal Shield West (BSW), and Boreal Shield East (BSE) ecozones. Variables listed include the Duff Moisture Code (DMC), Fine Fuel Moisture Code (FFMC), precipitation (PRECIP), number of lightning flashes (LDENS), ratio of precipitation to lightning flashes (DRYL), and day-of-year (jd). These models excluded the Drought Code variable

RF models consistently ranked DOY, DC, and DMC as the three most important variables contributing to lightning efficiency in all regions, with the exception of the SI and CI, based on both %IncMSE and IncNodePurity metrics (Fig. 7 and Fig. S11). In the SI, the FFMC and DRYL were also prominent, as were precipitation and DRYL in the CI depending on the importance metric. We also made regression models without the DC variable, because this variable was highly correlated (> 0.9) with DOY. And, as previously mentioned, correlations between DC and lightning efficiency showed relationships opposite to what we expected (i.e., lightning efficiency decreased as DC increased thereby indicating dryer conditions) for the BP, BSW, and BSE. The results for models excluding DC showed that DOY and DMC were still consistently the top two most important variables among regions with the exception of the SI where FFMC, DRYL, and precipitation were more prominent (Fig. 8 and Fig. S11). The percent variance explained by RF regression models was greatest for the SIM and MC, and was lowest for the CI and SI (Table 3). All regions showed a small decrease in variance explained when DC was removed from the RF models with the exception of the SI which showed an increase.

**Table 3:** Percent variance explained by Random Forest regression models predicting lightning efficiency (i.e., the ratio of cloud-to-ground lightning flashes per lightning-caused fire). Results include models both with and without the Duff Code (DC) variable as a predictor due to multicollinearity (see text)

Region	Variance explained (%) (with DC)	% Variance explained (%) (without DC)
*Ecozone*		
Montane Cordillera	53.66	51.20
Boreal Plains	50.45	49.57
Boreal Shield West	47.66	47.28
Boreal Shield East	48.37	45.17
*Ecoprovince*		
Southern Interior Mountains	54.11	52.72
Southern Interior	12.45	35.47
Central Interior	5.81	0.67

## Discussion

In this paper, we developed a climatology for lightning efficiency and fuel moisture variables over relatively broad regions of Canada (the ecozones), and a subset of regions in BC, that experienced the bulk of lightning-caused fires in the nation over our time series. Our results demonstrate that there are large-scale regional and temporal patterns in lightning efficiency (and, thus, the probability of a lightning flash igniting a wildfire) over the meteorological summer, which has important implications for wildfire modelling and management. For example, wildfire occurrence prediction models often include some measure of cloud-to-ground lightning activity as a covariate, for example, the number of flashes occurring in a spatiotemporal unit (e.g., Nadeem et al. 2020). Our results suggest that lightning-caused fire occurrence models may benefit from the addition of lightning covariates that are modified to account for spatiotemporal patterns in lightning efficiency. Our results for the BP (Fig. 2) illustrate how this may be useful in regions in which there is an asynchrony in the timing of peak lightning activity and peak wildland fire occurrence over the summer. Likewise, longitudinal differences in lightning efficiency over the summer also highlight this point, where the MC had the lowest lightning, and highest wildfire, density of the ecozones examined, and subsequently, the highest overall lightning efficiency. Localized spatiotemporal information related to lightning efficiency for individual areas and wildfires may be more useful for determining the likelihood of ignition and drivers of a particular fire than larger ecozone patterns; however, such information is often not available at localized spatiotemporal scales. Therefore, the broader regional summer patterns in lightning efficiency, and the relationships with soil moisture, presented herein are likely to be informative and may be applied more generally when finer resolution data is lacking. This assertion is supported by the fact that our findings are consistent with previously published research at more localized scales, which we further elaborate on below.

Related to our hypothesis, our correlation results suggested that in general the DMC and DC (however, see our discussion of DC below) were more highly correlated to lightning efficiency than the FFMC in most of the regions examined (an exception was the Central Interior). Being a surface layer, the FFMC is the most responsive fuel moisture variable of those we examined (e.g., its calculation includes wind speed as a parameter where the other deeper layers do not), and, as such, it is understandable that it showed the more variation over the summer than the DMC and DC. In fact, high inter-annual weather variability and resulting FFMC responses in the BSE and BSW may help explain the lack of clear seasonal patterns in lightning efficiency we found there. However, the FFMC has previously been shown to be important for lightning ignition in some regions. The research conducted by Nash and Johnson (1996), for example, found that the FFMC played an important role in lightning ignition efficiency in their Boreal forest study areas in Alberta and Saskatchewan. Likewise, Hessilt et al. (2022) found that the FFMC and DMC (which are more responsive to fire weather than the DC) were more influential for lightning efficiency than the DC which represents long-term drying of deep organic soils.

Our findings on the relative importance of DMC to lightning-caused fire are also consistent with other lightning-caused fire occurrence studies such as Wotton and Martell (2005) and Nadeem et al. (2020). One reason for this is likely due to the nature of lightning ignition: when a tree is struck by lightning, the electricity travels down the bole of the tree to the fuel layers below where ignition generally occurs (Latham and Williams 2001; Wotton et al. 2005). The ground fuel under a tree is typically sheltered by the canopy which intercepts precipitation (Wotton et al. 2005) — in fact, a modified version of the DMC that accounts for sheltering has been developed to account for this process (Wotton et al. 2005). Fires can combust and smolder in the deeper DMC layer at higher moisture contents than what would typically ignite the surface FFMC layer which is often wetter due to precipitation (Fransden 1997).

Our findings related to the seasonal increase in the DC over the summer are also consistent with previous research (McAlpine 1990). The relationships between DC and lightning efficiency (i.e., negative correlations with lightning efficiency ratio, and high importance in RF models) for the MC, SIM, SI, and CI is consistent with our expectations, where an increasing DC over the summer indicates long-term seasonal drying effects which could plausibly result in higher lightning efficiency. However, the relationships between DC and lightning efficiency in the BP, BSW, and BSE seem counterintuitive (i.e., that lightning efficiency decreases as long-term drying in deep soil layers increase) and may have more to do with the strong positive correlation between DC and DOY which was > 0.9 for all areas. Furthermore, based on previous research we have discussed, we did not expect the DC to perform so strongly compared to the other fuel moisture variables. As such, the importance of DC as a driver of lightning efficiency may be spurious in these cases because of the confounding influence of DOY. The DOY variable was consistently among the top three important drivers of lightning efficiency in RF models for ecozones and was the top variable in ecozone models where DC was removed. This may be because the DOY variable captures the influence of a number of unaccounted for environmental factors that change as the summer progresses. Such factors could include seasonal changes in above ground fuel phenology and characteristics including foliar moisture content and chemistry (Van Wagner 1967; Chrosciewicz 1986; Jolly et al. 2014). For instance, the efficiency patterns we observed in the BP and BSW, where peak efficiency occurred at the start of June and decreased through the summer, are consistent with seasonal patterns in green up and foliar moisture content (Pickell et al. 2017); in Alberta, for example, the spring-burning window refers to a period before green-up where vegetation is more susceptible to fire. Other seasonal factors are likely to be involved and should be included in future research.

Our findings are also consistent with previous research examining the ratio of lightning flashes to fires. For instance, Wierzchowski et al. (2002) examined the number of lightning discharges that occurred in the Central Cordillera ecosystem of BC and AB (their study area included parts of both the MC and BP) where their study area was roughly split in half by the continental divide. Their estimates of 1 fire for every 50 lightning discharges in the BC portion of their study area, and one fire for every 1400 lightning discharges on the Alberta side, is within the range of what we found in our study, despite using different methods, temporal period, and datasets. Likewise, Nausler (2014) found similar results in the western USA where they reported ~ 80,000 lightning strikes for 1910 wildfires (a ratio of ~ 42:1). Another study by Nash and Johnson (1996) examined 1,537,624 lightning strikes and 2551 fires (~ 602:1) in AB and Saskatchewan from May through August in four different years, which is similar to our results for the BSW. In Alaska and the Northwest Territories, between 2000 and 5000 cloud-to-ground flashes resulted in a fire (Hessilt et al. 2022). Similar findings have also been reported outside of North America. For example, in Catalonia, Spain, 1 in 1000 cloud-to-ground lightning flashes was reported to have resulted in a wildfire (Soler et al. 2021). Human influences on fuels can also play a role, as Meisner (1993) found lightning efficiency to vary by fuel type in Southern Idaho, where the highest efficiency occurred in logging slash (10:1) and the lowest in agricultural crops (approximately 333:1). The spatial differences in lightning efficiency between the ecozones we examined could be influenced by several factors associated with the regions including the types of fuel present there. Coniferous trees are typically more flammable and receptive to ignition than deciduous trees (Krawchuk et al. 2006; Rogers et al. 2015; Hessilt et al. 2022). The MC contains a higher proportion of coniferous fuels than the BP, BSE, and BSW which contain higher proportions of deciduous fuels (Wotton et al. 2017) which may help explain the higher lightning efficiency we observed there. Synoptic weather patterns have also been shown to influence lightning efficiency (Nash and Johnson 1996). The increase in lightning efficiency over the summer in the MC is likely influenced by the stationary high-pressure ridge that tends to form over the province that can result in prolonged periods of clear, dry, warm weather and that dry out fuels over the summer (Johnson et al. 1990). Variations in topography (which are most variable in the MC) can also affect forest cover and fuel moisture content and thus lightning ignition (Dissing and Verbyla 2003; Krawchuk et al. 2006).

The BC ecoprovinces experienced a higher density of lightning-caused fires compared to the ecozones. Of the BC ecoprovinces (which are subareas of the MC), the SI had the highest lightning efficiency, while the overall MC had the lowest density of lightning flashes and highest density of fires of all regions examined. The MC lies in the rain shadow of the Coast and Cascade Mountains and contains some of the hottest and driest areas in the BC. The SI is also home to ponderosa pine (*Pinus ponderosa*) forests which are highly flammable and have a short fire return interval (Fonda 2001). The SIM also had a relatively high maximum lightning efficiency (lower than in the SI, but higher than for the overall MC) and a low predicted efficiency at the start of the summer. However, the SIM showed the highest correlation with all three fuel moisture variables than any other area examined, and also displayed the highest correlations with precipitation and dry lightning. The SIM also had the highest variance explained by RF models than in other areas, highlighting the importance of these variables in this mountainous region. In the CI, maximum lightning efficiency was lower than for the other ecoprovinces and for the MC as a whole; however, the minimum predicted lightning efficiency in early June was higher than for the SIM and MC. However, both the linear and RF model for the CI performed relatively poorly, suggesting that other variables may be more strongly influencing lightning efficiency in this ecoprovince.

The characteristics of the cloud-to-ground lightning flashes in different ecozones, the influence of polarity, amplitude, and multiplicity on ignition efficiency are not fully understood. For instance, research has shown conflicting results regarding the probability of a positively versus negatively charged flash starting a fire (Latham and Schlieter 1989; Flannigan and Wotton 1990). We note that recent research in Alaska and the Northwest Territories found that ~ 85% of fires were caused by negatively charged flashes which was in proportion to the overall distribution of lightning flash charges that occurred there (Hessilt et al. 2022). We found similar proportions of negatively charged flashes in Canadian ecozones (range 80.5–86.8).

### Limitations and future work

A main intent of this paper was to focus on the relative importance of different fuel moisture variables on lightning ignition efficiency as such variables have been shown to be important for lightning ignition and efficiency in previous research; however, we recognize that there are a number of other variables which could be examined in relation to lightning efficiency in future work. Likewise, lightning efficiency research should continue in other regions and at finer scales to uncover patterns and processes related to lightning efficiency at more localized scales.

There are also some limitations when using the agency report day for the start of a fire (as was reported for the NFDB point dataset we used), because there can be a lag between the time a fire started and the time it was detected. For example, lightning-caused fire can smolder in the DMC for several days before “arriving” and being detected by fire management agencies and remote sensing platforms (Wotton et al. 2005). This limitation could be addressed in more localized modelling where spatiotemporal lightning strike patterns can be analyzed in relation to individual fires to identify the most likely individual fire-causing flashes when data is available. One thing to note is that our estimates of lightning efficiency are conservative in that they include the total amount of lightning in each study area (as is commonly done) without considering the fact that cloud-to-ground lightning data in the CLDN includes strikes that occur over water bodies and areas of non-fuels. Thus, filtering out cloud-to-ground strikes that occur on areas of non-fuels could be beneficial, but one would also need to consider any error associated with lightning flash locations.

A limitation of our variable DRYL is that it does not capture the dry lightning conditions at a particular fire, which may be a reason why we found lower performance for this variable in some areas; however, it does give an idea of the amount of precipitation that accompanies lightning on different days over the summer in a particular region and can therefore provide insight into this type of seasonality. Dry lightning has been to shown to be a strong factor for lightning-caused fires in some areas (e.g., Wickramasinghe et al. 2024), and dry lightning has been shown to occur more frequently at higher elevations during the summer months in California, USA (Kalashnikov et al. 2022). Interestingly, we found that the DRYL variable had the highest correlation with lightning efficiency in the Southern Interior Mountains ecoprovince which contains large mountain ranges.

### Climate change

It is very likely that patterns in lightning efficiency will respond to the effects of climate change, because changes in vegetation and lightning are expected as the climate continues to shift (Veraverbeke et al 2017; Stralberg et al. 2018; Bieniek et al. 2020; Chen et al. 2021; Hessilt et al. 2022). For instance, Romps et al. (2014) predicted an approximately 50% increase in lightning in the contiguous USA over the current century. Conversely, some research has projected a global decrease in lightning as the climate changes; however, the projected decreases occur primarily over the tropics while large regions of Canada, particularly in the north, are predicted to see an increase in lightning flash rate over the century (Finney et al. 2018). Recent research using Environment and Climate Change Canada’s global atmospheric model (CanAM5.1) reports a similar message, as they predict that increases in lightning in northern mid-latitudes will result in greater area burned by the end of the century (Whaley et al. 2024). Changes in the distribution and type of wildfire fuels are also expected in response the combined effects of climate change and changes in fire regimes (Stralberg et al. 2018). Such changes in lightning and fuel patterns would thus logically lead to alterations in spatiotemporal patterns of lightning efficiency in the future. Thus, ongoing research aimed at improving the understanding of lightning efficiency will be important going forward, while this and previous research can help to establish benchmarks for recent times.

## Supplementary Information

Supplementary Material 1.

## Data Availability

Wildfire data used in this study is available online at https://cwfis.cfs.nrcan.gc.ca/datamart. ERA5 data used in this study is available online at https://www.ecmwf.int/en/forecasts/dataset/ecmwf-reanalysis-v5. Lightning data used in this study is available from Vaisala (https://www.vaisala.com/en).

## Abbreviations

%IncMSE: Percent increase in Mean Squared Error
BC: British Columbia
BP: Boreal Plains Ecozone
BSE: Boreal Shield East Ecozone
BSW: Boreal Shield West Ecozone
CI: Central Interior Ecoprovince
DC: Drought Code
DMC: Duff Moisture Code
DOY: Day of year
DRYL: Average precipitation (mm) divided by the number of lightning flashes
FFMC: Fine Fuel Moisture Code
FWI System: Canadian Forest Fire Weather Index System
IncNodePurity: Increasing Node Purity
MC: Montane Cordillera Ecozone
RF: Random Forest regression model
R_s_: Spearman’s rho
SI: Southern Interior Ecoprovince
SIM: Southern Interior Mountains Ecoprovince
