# Supplementary material for: Lightning ignition efficiency in Canadian forests
Source: Fire Ecol. 2025 May 26;21(1):34. doi: 10.1186/s42408-025-00376-1 (PMC12104117; doi:10.1186/s42408-025-00376-1)
Supplement: Supplementary file 1 — Supplementary Material 1. [file 42408_2025_376_MOESM1_ESM.zip › Coogan_FireEcology_SUPPLEMENTARY_MATERIAL.pdf]

Supplementary Material

```
Call:
lm(formula = EF ~ jd + jd2)

Residuals:
     Min       1Q   Median       3Q      Max 
-184.924  -47.143   -8.919   43.516  248.556 

Coefficients:
             Estimate Std. Error t value Pr(>|t|)    
(Intercept) 381.76988   24.29130  15.716  < 2e-16 ***
jd           -8.78785    1.21871  -7.211 1.84e-10 ***
jd2           0.06408    0.01284   4.993 2.98e-06 ***
---
Signif. codes:  0 '***' 0.001 '**' 0.01 '*' 0.05 '.' 0.1 ' ' 1

Residual standard error: 75.55 on 88 degrees of freedom
Multiple R-squared:  0.5706,	Adjusted R-squared:  0.5608 
F-statistic: 58.47 on 2 and 88 DF,  p-value: < 2.2e-16
```

Fig. S1 Linear model summary for daily lightning ignition efficiency in the Montane Cordillera Ecozone as displayed in the RStudio for the R statistical software environment.

```
Call:
lm(formula = EF_BP ~ jd_BP)

Residuals:
   Min     1Q Median     3Q    Max 
-960.9 -322.2 -109.6  136.6 2133.5 

Coefficients:
            Estimate Std. Error t value Pr(>|t|)    
(Intercept)  373.787    118.937   3.143  0.00227 ** 
jd_BP         14.635      2.245   6.518 4.17e-09 ***
---
Signif. codes:  0 '***' 0.001 '**' 0.01 '*' 0.05 '.' 0.1 ' ' 1

Residual standard error: 562.6 on 89 degrees of freedom
Multiple R-squared:  0.3231,	Adjusted R-squared:  0.3155 
F-statistic: 42.48 on 1 and 89 DF,  p-value: 4.168e-09
```

Fig. S2 Linear model summary for daily lightning ignition efficiency in the Boreal Plains Ecozone as displayed in the RStudio for the R statistical software environment.

```
Call:
lm(formula = EF_BSW ~ jd_BSW)

Residuals:
    Min      1Q  Median      3Q     Max 
-388.81 -120.87   -9.41  118.05  489.29 

Coefficients:
            Estimate Std. Error t value Pr(>|t|)    
(Intercept) 446.7511    38.7301  11.535  < 2e-16 ***
jd_BSW        2.0441     0.7311   2.796  0.00634 ** 
---
Signif. codes:  0 ‘***’ 0.001 ‘**’ 0.01 ‘*’ 0.05 ‘.’ 0.1 ‘ ’ 1

Residual standard error: 183.2 on 89 degrees of freedom
Multiple R-squared:  0.08073,	Adjusted R-squared:  0.0704 
F-statistic: 7.816 on 1 and 89 DF,  p-value: 0.006344
```

Fig. S3 Linear model summary for daily lightning ignition efficiency in the Boreal Shield West Ecozone as displayed in the RStudio for the R statistical software environment.

```
Call:
lm(formula = EF_BSEout ~ jd_BSEout)

Residuals:
    Min      1Q  Median      3Q     Max 
-705.16 -309.17  -78.22  223.49 1373.57 

Coefficients:
            Estimate Std. Error t value Pr(>|t|)    
(Intercept)  775.658     96.288   8.056 3.81e-12 ***
jd_BSEout      1.733      1.820   0.952    0.344    
---
Signif. codes:  0 ‘***’ 0.001 ‘**’ 0.01 ‘*’ 0.05 ‘.’ 0.1 ‘ ’ 1

Residual standard error: 447.1 on 87 degrees of freedom
Multiple R-squared:  0.01032,	Adjusted R-squared:  -0.001059 
F-statistic: 0.9069 on 1 and 87 DF,  p-value: 0.3436
```

Fig. S4A Linear model summary for daily lightning ignition efficiency in the Boreal Shield East Ecozone as displayed in the RStudio for the R statistical software environment. Two outliers were removed from the data set. This model was used in the paper.

```
Call:
lm(formula = EF_BSE ~ jd_BSE)

Residuals:
   Min     1Q Median     3Q    Max 
-784.0 -394.7 -161.8  164.9 4317.1 

Coefficients:
            Estimate Std. Error t value Pr(>|t|)    
(Intercept)  853.232    156.624   5.448 4.49e-07 ***
jd_BSE         1.952      2.957   0.660    0.511    
---
Signif. codes:  0 ‘***’ 0.001 ‘**’ 0.01 ‘*’ 0.05 ‘.’ 0.1 ‘ ’ 1

Residual standard error: 740.9 on 89 degrees of freedom
Multiple R-squared:  0.004875,	Adjusted R-squared:  -0.006306 
F-statistic: 0.436 on 1 and 89 DF,  p-value: 0.5108
```

Fig. S4B Linear model summary for daily lightning ignition efficiency in the Boreal Shield East Ecozone, including the two outliers that were removed, as displayed in the RStudio for the R statistical software environment. This model was not used in the paper, but is shown here for comparison.

```
Call:
lm(formula = EF_SIM ~ jd_SIM + jd2_SIM)

Residuals:
    Min      1Q  Median      3Q     Max 
-205.04  -41.03  -12.71   31.03  290.34 

Coefficients:
              Estimate Std. Error t value Pr(>|t|)    
(Intercept) 3683.91105  526.73783   6.994 4.98e-10 ***
jd_SIM       -32.76900    5.38561  -6.085 2.96e-08 ***
jd2_SIM        0.07388    0.01358   5.442 4.69e-07 ***
---
Signif. codes:  0 ‘***’ 0.001 ‘**’ 0.01 ‘*’ 0.05 ‘.’ 0.1 ‘ ’ 1

Residual standard error: 79.91 on 88 degrees of freedom
Multiple R-squared:  0.6315,	Adjusted R-squared:  0.6231 
F-statistic:  75.4 on 2 and 88 DF,  p-value: < 2.2e-16
```

Fig. S5 Linear model summary for daily lightning ignition efficiency in the Southern Interior Mountains Ecoprovince as displayed in the RStudio for the R statistical software environment.

```
Call:
lm(formula = EF_SIout2 ~ jd_SIout2 + jd2_SIout2)

Residuals:
    Min      1Q  Median      3Q     Max 
-68.554 -24.346  -8.004  14.962 184.182 

Coefficients:
              Estimate Std. Error t value Pr(>|t|)   
(Intercept) 965.570187 293.085507   3.294  0.00143 **
jd_SIout2    -7.998356   2.995682  -2.670  0.00907 **
jd2_SIout2    0.017206   0.007554   2.278  0.02521 * 
---
Signif. codes:  0 ‘***’ 0.001 ‘**’ 0.01 ‘*’ 0.05 ‘.’ 0.1 ‘ ’ 1

Residual standard error: 42.52 on 86 degrees of freedom
Multiple R-squared:  0.3728,	Adjusted R-squared:  0.3582 
F-statistic: 25.56 on 2 and 86 DF,  p-value: 1.946e-09
```

Fig. S6A Linear model summary for daily lightning ignition efficiency in the Southern Interior Ecoprovince as displayed in the RStudio for the R statistical software environment.

```
Call:
lm(formula = EF_SIout ~ jd_SIout + jd2_SIout)

Residuals:
   Min     1Q Median     3Q    Max 
-75.60 -31.69 -10.88  14.58 291.99 

Coefficients:
              Estimate Std. Error t value Pr(>|t|)    
(Intercept)  1.379e+03  3.615e+02   3.815 0.000255 ***
jd_SIout    -1.244e+01  3.687e+00  -3.373 0.001110 ** 
jd2_SIout    2.897e-02  9.276e-03   3.123 0.002429 ** 
---
Signif. codes:  0 ‘***’ 0.001 ‘**’ 0.01 ‘*’ 0.05 ‘.’ 0.1 ‘ ’ 1

Residual standard error: 53.49 on 87 degrees of freedom
Multiple R-squared:  0.2477,	Adjusted R-squared:  0.2304 
F-statistic: 14.33 on 2 and 87 DF,  p-value: 4.187e-06
```

Fig. S6B Linear model summary for daily lightning ignition efficiency in the Southern Interior Ecoprovince, including the two outliers that were removed, as displayed in the RStudio for the R

statistical software environment. This model was not used in the paper, but is shown here for comparison.

```
Call:
lm(formula = EF_CI ~ jd_CI + jd_CI2)

Residuals:
    Min      1Q  Median      3Q     Max
-162.85  -58.31  -13.45   38.66  420.56

Coefficients:
              Estimate Std. Error t value Pr(>|t|)
(Intercept) 2468.54619  659.50800   3.743 0.000324 ***
jd_CI        -22.58103    6.74311  -3.349 0.001196 **
jd_CI2         0.05405    0.01700   3.180 0.002035 **
---
Signif. codes:  0 ‘***’ 0.001 ‘**’ 0.01 ‘*’ 0.05 ‘.’ 0.1 ‘ ’ 1

Residual standard error: 100.1 on 88 degrees of freedom
Multiple R-squared:  0.176,	Adjusted R-squared:  0.1572
F-statistic: 9.396 on 2 and 88 DF,  p-value: 0.0002002
```

Fig. S7 Linear model summary for daily lightning ignition efficiency in the Central Interior Ecoprovince as displayed in the RStudio for the R statistical software environment.

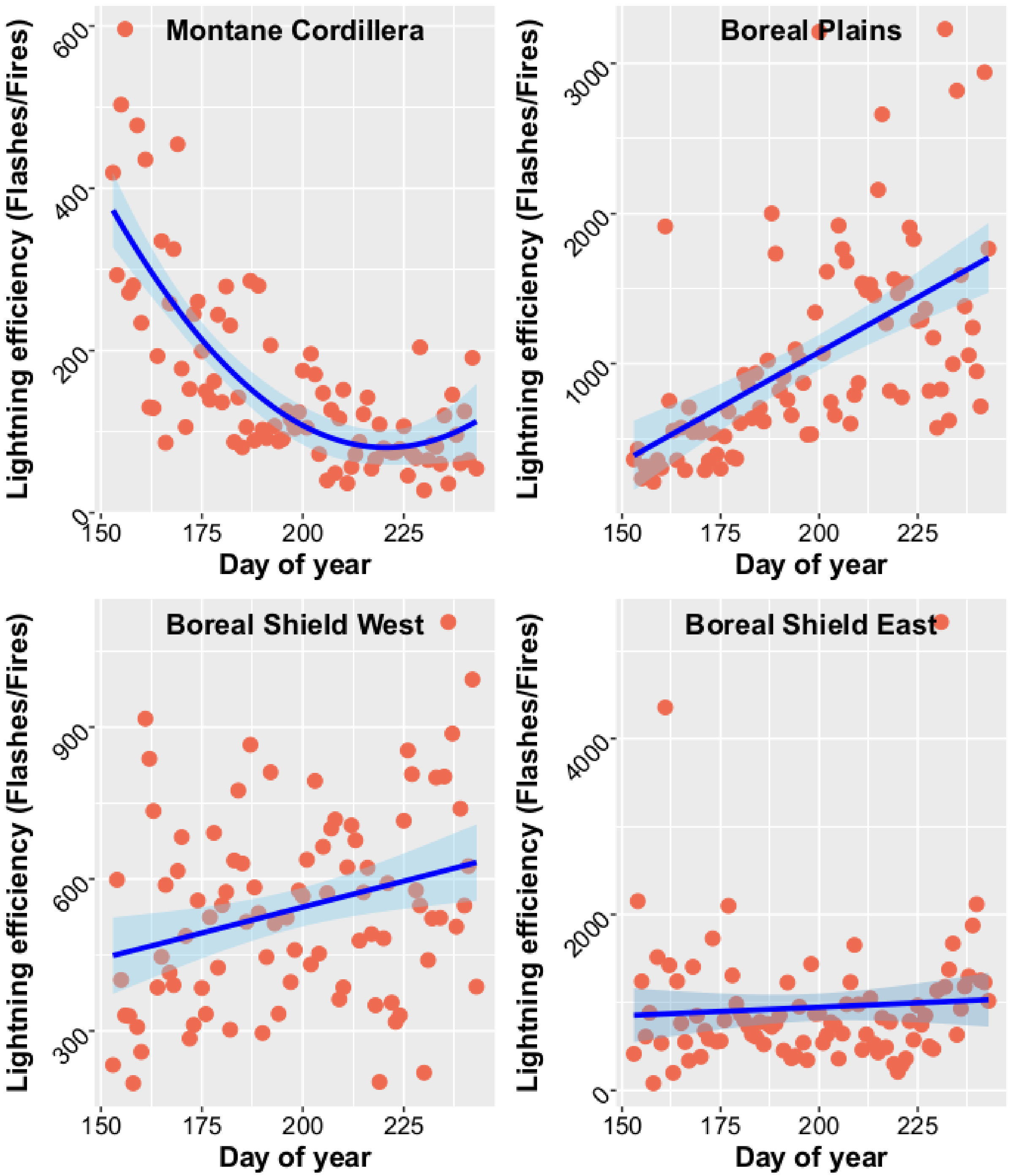

Fig. S8 Daily lightning efficiency (i.e., the ratio of cloud-to-ground lightning flashes per lightning-caused fire) for the Montane Cordillera, Boreal Plains, Boreal Shield West, and Boreal Shield East Ecozones over the meteorological summer (2001-2020). The two outliers that were

removed from the Boreal Shield East Ecozone analysis are included in this figure. All other figure panels are the same as presented in the main paper.

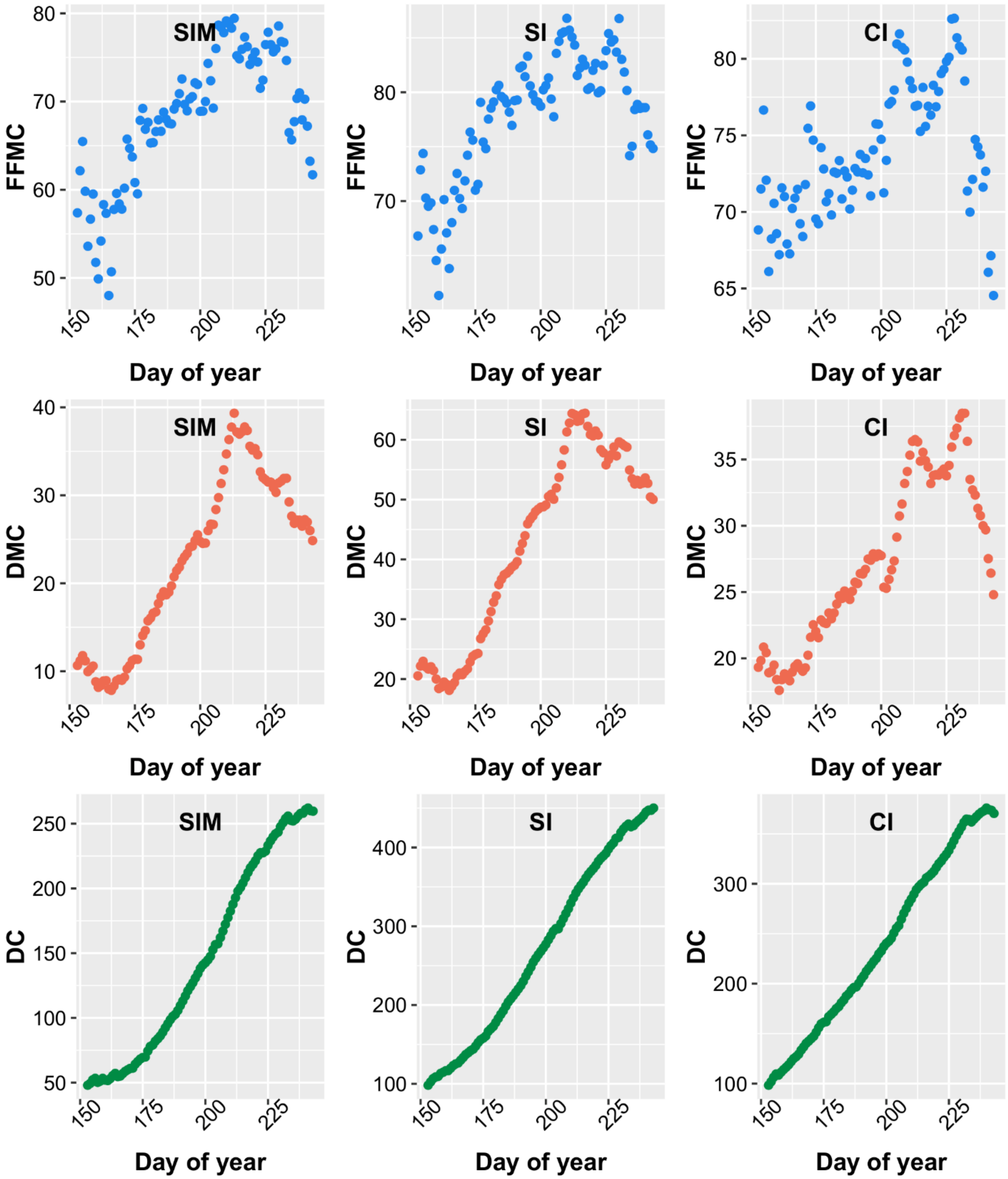

Fig. S9 Average daily values (2001–2020) for the Fine Fuel Moisture Code (FFMC; top row), Duff Moisture Code (DMC; middle row), and Duff Code (DC; bottom row) for the Southern Interior Mountains (SIM), Southern Interior (SI), and Central Interior (CI) Ecoprovinces over the meteorological summer (June – August).

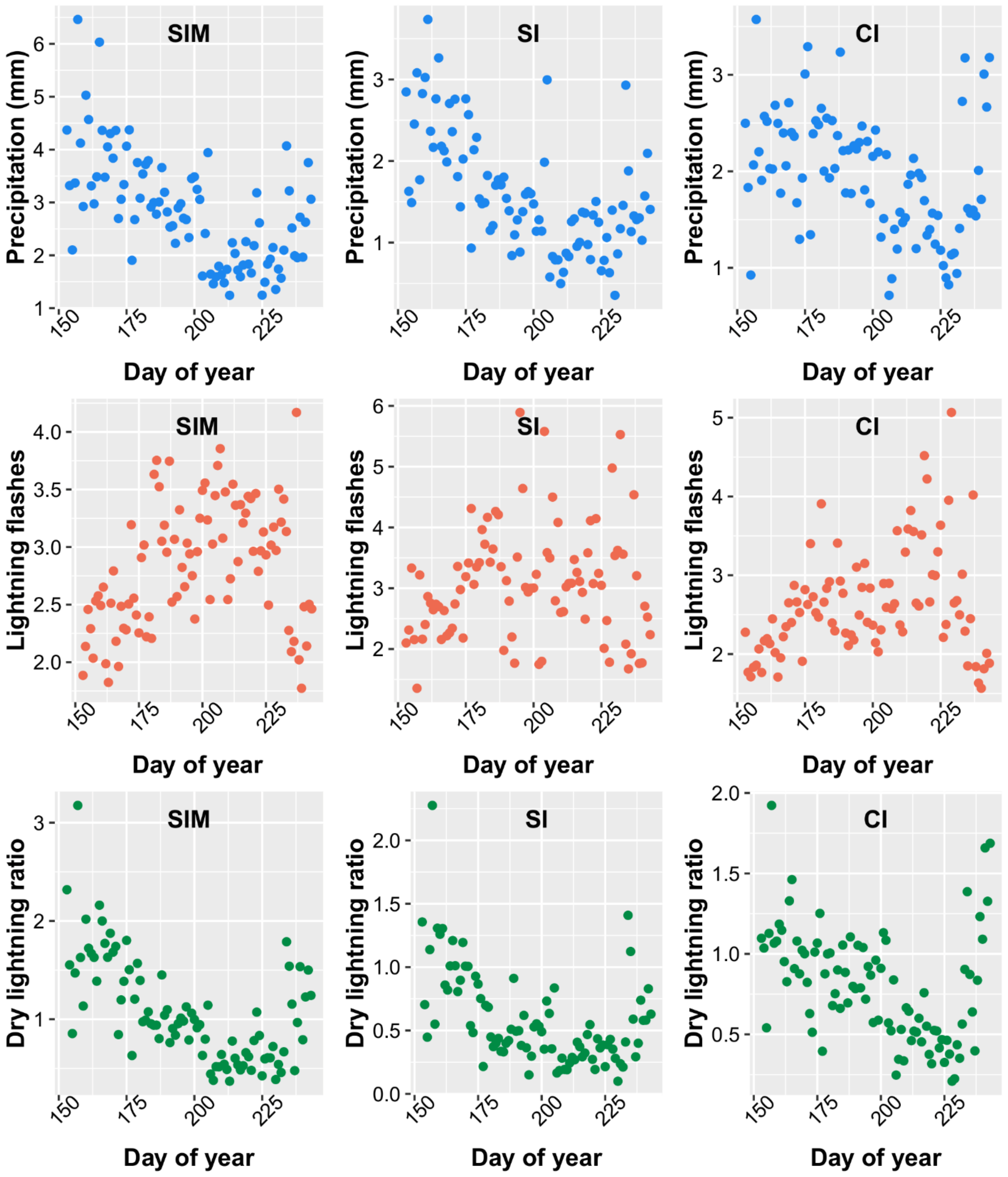

Fig. S10 Average daily values (2001–2020) For precipitation (mm; top row), number of cloud-to-ground lightning flashes (middle row), and dry lightning ratio (i.e., the ratio of precipitation to

flashes; bottom row) for the Southern Interior Mountains (SIM), Southern Interior (SI), and Central Interior (CI) Ecoprovinces over the meteorological summer (June – August).

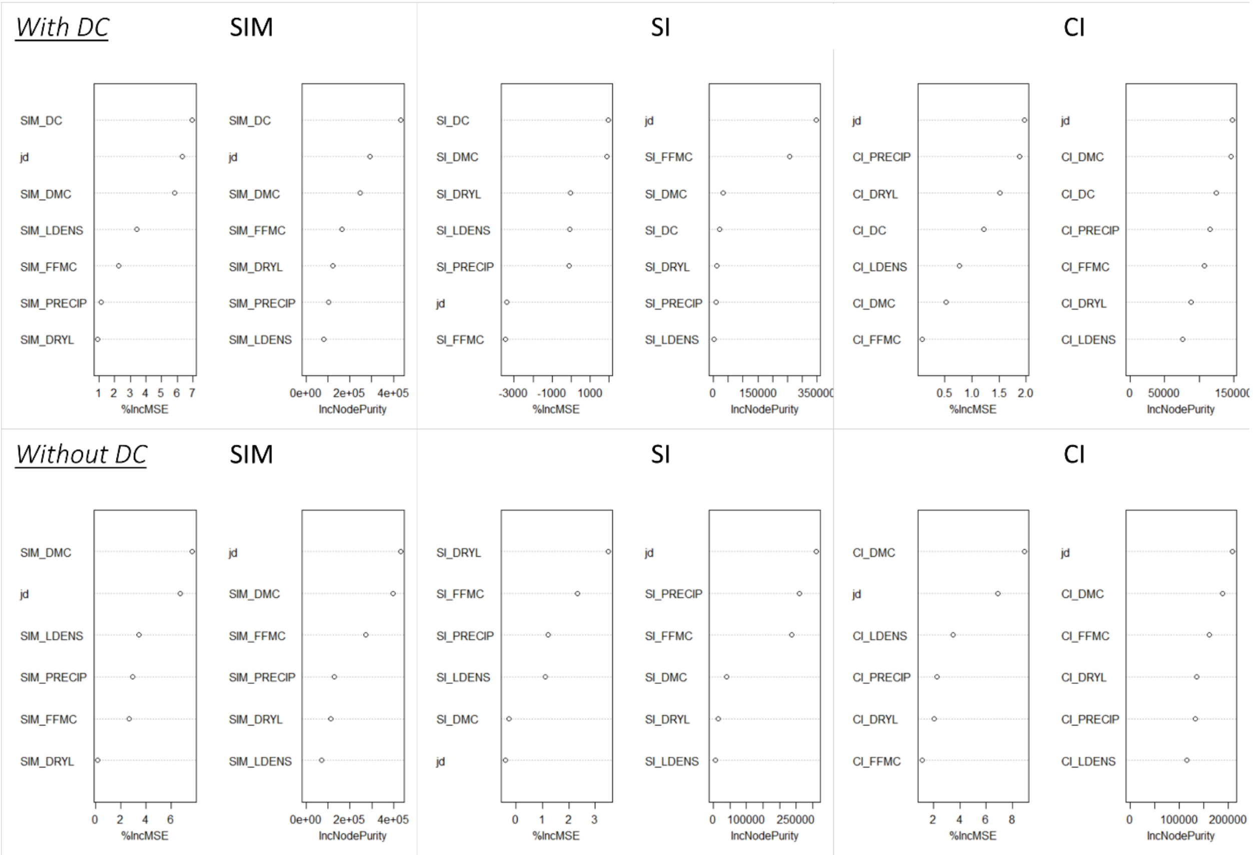

Fig. S11 Variable importance for Random Forest regression models, including both the percent increase in mean squared error (%IncMSE) and increasing node purity (IncNodePurity) metrics, for the Southern Interior Mountains (SIM), Southern Interior (SI), and Central Interior (CI) ecoprovinces. Variables listed include the Drought Code (DC; top row only), Duff Moisture Code (DMC), Fine Fuel Moisture Code (FFMC), precipitation (PRECIP), number of lightning flashes (LDENS), ratio of precipitation to lightning flashes (DRYL), and day-of-year (jd).

Time series adjustment for leap years

We modified our time series slightly to account for the effect of leap years (n=6; i.e., 2000, 2004, 2008, 2012, 2016, 2020) on the DOY number assignment. For non-leap years, DOYs ranged from 152–243 for the meteorological summer (i.e., 01 June to 31 August). For leap years, however, the DOYs for the summer ranged from 153–244 because of the addition of an extra day earlier in the year (i.e., 29 February). Thus, the DOYS in the time series ranged from 152-244, where DOY 152 had only 15 observations (21 years of data for the time series minus 6 leap years where 01 July was on DOY 153) and DOY 244 had only 6 observations (i.e., DOY 244 corresponds to 31 August for the leap years, but corresponds to 01 September for the non-leap years). Rather than performing an offset to the leap year DOYs (by subtracting 1 from the leap year DOYs), which is one approach to this problem, we simply truncated the data set by removing DOYS 152 and 244 from our analysis; thus, our time series ran from 02 June to 31 August with the full number of yearly observations across the timeseries.
